# Supplementary material for: Drone-based effective counting and ageing of hippopotamus (Hippopotamus amphibius) in the Okavango Delta in Botswana
Source: PLoS One. 2019 Dec 5;14(12):e0219652. doi: 10.1371/journal.pone.0219652 (PMC6894862; doi:10.1371/journal.pone.0219652)
Supplement: S1 Text — (DOCX) [file pone.0219652.s002.docx]

**S1 Text. Results of statistical models, as output from R.**

Effect of survey height, time of day, their interaction, survey date, and inclusion of an AR(1) covariance structure on variations in total hippo count (model 1), percentage of hippos assigned to age classes (number of juveniles, subadults, and adults divided by the total count for each drone/land survey; model 2), and counts of juveniles, subadults, and adults (models 3 - 5) using glmmTMB function (glmmTMBpackage). Where models showed no significant interaction effect and no significant improvement with the inclusion of an AR(1) covariance structure, models was rerun removing these variables. Significance of effects was tested using likelihood ratio tests by comparing full and reduced models with the anova function. Pairwise comparisons of effect of time of day and height were conducted using the emmeans function (emmeans package).

**Table A. Model 1 - Total hippo count**

| **Effect of inclusion of AR(1) variable** | | | | | | | | | | | | | |
| --- | --- | --- | --- | --- | --- | --- | --- | --- | --- | --- | --- | --- | --- |
| Analysis of Variance Table using likelihood ratio tests | | | | | | | | | | | | | |
| Mod.AR1=glmmTMB(total.count ~ height*time + (1\|date) + ar1(times+0\|group)) | | | | | | | | | | | | | |
| Mod =glmmtTMB(total.count ~ height*time + (1\|date)) | | | | | | | | | | | | | |
|  | Df | | AIC | | BIC | logLik | | | Dev | Chisq | | Pr(>Chisq) | |
| Mod | 18 | | 467.64 | | 516.58 | -215.82 | | | 431.64 |  | |  | |
| Mod.AR1 | 20 | | 468.10 | | 522.47 | -214.05 | | | 428.10 | 3.548 | | 0.170 | |
|  |  | |  | |  |  | | |  |  | |  | |
| **Effect of interaction between time and height** | | | | | | | | | | | | | |
| Analysis of Variance Table using likelihood ratio tests | | | | | | | | | | | | | |
| Full.mod.int = glmmTMB(total.count ~ height * time + (1\|date)) | | | | | | | | | | | | | |
| Full.mod = glmmtTMB(total.count ~ height + time + (1\|date)) | | | | | | | | | | | | | |
|  | Df | | AIC | | BIC | logLik | | | Dev | Chisq | | Pr(>Chisq) | |
| Full.mod | 9 | | 460.92 | | 485.39 | -221.46 | | | 442.92 |  | |  | |
| Full.mod.int | 18 | | 467.64 | | 516.58 | -215.82 | | | 431.64 | 11.276 | | 0.257 | |
|  |  | |  | |  |  | | |  |  | |  | |
| **Effect of height** | | | | | | | | | | | | | |
| Analysis of Variance Table using likelihood ratio tests | | | | | | | | | | | | | |
| Full.mod = glmmTMB(total.count ~ height + time + (1\|date)) | | | | | | | | | | | | | |
| Red.mod = glmmtTMB(total.count ~ time + (1\|date)) | | | | | | | | | | | | | |
|  | Df | | AIC | | BIC | logLik | | | Dev | Chisq | | Pr(>Chisq) | |
| Red.mod | 6 | | 467.10 | | 483.41 | -227.55 | | | 455.10 |  | |  | |
| Full.mod | 9 | | 460.92 | | 485.39 | -221.46 | | | 442.92 | 12.180 | | 0.007** | |
|  |  | |  | |  |  | | |  |  | |  | |
| **Effect of time** | | | | | | | | | | | | | |
| Analysis of Variance Table using likelihood ratio tests | | | | | | | | | | | | | |
| Full.mod = glmmTMB(total.count ~ height + time + (1\|date)) | | | | | | | | | | | | | |
| Red.mod = glmmtTMB(total.count ~ height + (1\|date)) | | | | | | | | | | | | | |
|  | Df | | AIC | | BIC | logLik | | | Dev | Chisq | | Pr(>Chisq) | |
| Red.mod | 6 | | 493.30 | | 509.61 | -240.65 | | | 481.30 |  | |  | |
| Full.mod | 9 | | 460.92 | | 485.39 | -221.46 | | | 442.92 | 38.384 | | <0.001*** | |
|  |  | |  | |  |  | | |  |  | |  | |
| **Effect of date (random effect)** | | | | | | | | | | | | | |
| Analysis of Variance Table using likelihood ratio tests | | | | | | | | | | | | | |
| Full.mod = glmmTMB(total.count ~ height + time + (1\|date)) | | | | | | | | | | | | | |
| Red.mod = glmmtTMB(total.count ~ height + time) | | | | | | | | | | | | | |
|  | Df | | AIC | | BIC | logLik | | | Dev | Chisq | | Pr(>Chisq) | |
| Red.mod | 8 | | 523.68 | | 545.43 | -253.84 | | | 507.68 |  | |  | |
| Full.mod | 9 | | 460.92 | | 485.39 | -221.46 | | | 442.92 | 64.757 | | <0.001*** | |
|  |  | |  | |  |  | | |  |  | |  | |
| **Emmeans and pairwise differences (height)** | | | | | | | | | | | | | |
| emmeans(Full.mod, list(pairwise ~ height), adjust = tukey) | | | | | | | | | | | | | |
| height | | emmean | | SE | | | df | lower.CL | | | upper.CL | |  |
| Land count | | 9.07 | | 0.711 | | | 103 | 7.66 | | | 10.48 | |  |
| 40 | | 10.04 | | 0.711 | | | 103 | 8.62 | | | 11.45 | |  |
| 80 | | 9.07 | | 0.711 | | | 103 | 7.66 | | | 10.48 | |  |
| 120 | | 8.54 | | 0.711 | | | 103 | 7.12 | | | 9.95 | |  |
|  | |  | |  | | |  |  | | |  | |  |
| contrast | | | | estimate | | | SE | df | | | t.ratio | | p.value |
| Land count - 40 | | | | -0.964 | | | 0.426 | 103 | | | 2.264 | | 0.113 |
| Land count - 80 | | | | 0.000 | | | 0.426 | 103 | | | 0.000 | | 1.000 |
| Land count - 120 | | | | 0.536 | | | 0.426 | 103 | | | 1.258 | | 0.592 |
| 40 - 80 | | | | 0.964 | | | 0.426 | 103 | | | 2.264 | | 0.113 |
| 40 – 120 | | | | 1.500 | | | 0.426 | 103 | | | 3.522 | | 0.004** |
| 80 - 120 | | | | 0.536 | | | 0.426 | 103 | | | 1.258 | | 0.592 |
| Results are averaged over the levels of: time  Degrees-of-freedom method: kenward-roger  Confidence level used: 0.95  P value adjustment: tukey method for comparing family of 4 estimates | | | | | | | | | | | | | |
| Results are averaged over the levels of: time  Confidence level used: 0.95  P value adjustment: tukey method for comparing family of 4 estimates | | | | | | | | | | | | | |
| **Emmeans and pairwise differences (time)** | | | | | | | | | | | | | |
| emmeans(Full.mod, list(pairwise ~ time), adjust = tukey) | | | | | | | | | | | | | |
| time | | emmean | | SE | | | df | lower.CL | | | upper.CL | |  |
| EM | | 7.43 | | 0.711 | | | 103 | 6.02 | | | 8.84 | |  |
| LM | | 9.68 | | 0.711 | | | 103 | 8.27 | | | 11.09 | |  |
| EA | | 10.04 | | 0.711 | | | 103 | 8.62 | | | 11.45 | |  |
| LA | | 9.57 | | 0.711 | | | 103 | 8.16 | | | 10.98 | |  |
|  | |  | |  | | |  |  | | |  | |  |
| contrast | estimate | | | SE | | | df | t.ratio | | | p.value | |  |
| EM – LM | -2.250 | | | 0.426 | | | 103 | -5.284 | | | <0.001*** | | |
| EM – EA | -2.607 | | | 0.426 | | | 103 | -6.122 | | | <0.001*** | | |
| EM – LA | -2.143 | | | 0.426 | | | 103 | -5.032 | | | <0.001*** | | |
| LM – EA | -0.357 | | | 0.426 | | | 103 | -0.839 | | | 0.836 | |  |
| LM – LA | 0.107 | | | 0.426 | | | 103 | 0.252 | | | 0.994 | |  |
| EA - LA | 0.464 | | | 0.426 | | | 103 | 1.090 | | | 0.696 | |  |
|  | | | | | | | | | | | | | |
| Results are averaged over the levels of: height  Confidence level used: 0.95  P value adjustment: tukey method for comparing family of 4 estimates | | | | | | | | | | | | | |

**Table B. Model 2 - Percentage of hippos assigned to age classes**

| **Effect of inclusion of AR(1) variable** | | | | | | | | | | | | | | | |
| --- | --- | --- | --- | --- | --- | --- | --- | --- | --- | --- | --- | --- | --- | --- | --- |
| Analysis of Variance Table using likelihood ratio tests | | | | | | | | | | | | | | | |
| Mod.AR1 = glmmTMB(percentage ~ height*time + (1\|date)  xfchbxfdghfgh  ) | | | | | | | | | | | | | | | |
| + ar1(times+0\|group), family=binomial, weights=total)  weights=total) | | | | | | | | | | | | | | | |
| Mod = glmmTMB(percentage ~ height*time + (1\|date), | | | | | | | | | | | | | | | |
| family=binomial, weights=total | | | | | | | | | | | | | | | |
|  | Df | AIC | | BIC | | logLik | | | Dev | | | Chisq | | | Pr(>Chisq) |
| Mod | 17 | 463.07 | | 509.29 | | -214.54 | | | 429.07 | | |  | | |  |
| Mod.AR1 | 19 | 448.31 | | 499.97 | | -205.16 | | | 410.31 | | | 18.760 | | | <0.001*** |
|  |  |  | |  | |  | | |  | | |  | | |  |
| **Effect of interaction between time and height** | | | | | | | | | | | | | | | |
| Analysis of Variance Table using likelihood ratio tests | | | | | | | | | | | | | | | |
| Full.mod = glmmTMB(percentage ~ height*time + (1\|date) | | | | | | | | | | | | | | | |
| + ar1(times+0\|group),family=binomial, weights=total) | | | | | | | | | | | | | | | |
| Full.mod.add = glmmTMB(percentage ~ height+time + (1\|date) | | | | | | | | | | | | | | | |
| + ar1(times+0\|group), family=binomial, weights=total) | | | | | | | | | | | | | | | |
|  | | Df | AIC | | BIC | | logLik | | | | Dev | | Chisq | | Pr(>Chisq) |
| Full.mod.add | | 10 | 447.42 | | 474.61 | | -213.71 | | | | 427.42 | |  | |  |
| Full.mod | | 19 | 448.31 | | 499.97 | | -205.16 | | | | 410.31 | | 17.10008 | | 0.047* |
|  | |  |  | |  | |  | | | |  | |  | |  |
| **Effect of date (random effect)** | | | | | | | | | | | | | | | |
| Analysis of Variance Table using likelihood ratio tests | | | | | | | | | | | | | | | |
| Full.mod = glmmTMB(percentage ~ height*time + (1\|date)  + ar1(times+0\|group), family=binomial, weights=total) | | | | | | | | | | | | | | | |
| Red.mod = glmmTMB(percentage ~ height*time + ar1(times+0\|group), | | | | | | | | | | | | | | | |
| family=binomial, weights=total) | | | | | | | | | | | | | | | |
|  | Df | AIC | | BIC | | logLik | | | Dev | | | Chisq | | | Pr(>Chisq) |
| Red.mod | 18 | 446.32 | | 495.25 | | -205.16 | | | 410.32 | | |  | | |  |
| Full.mo | 19 | 448.31 | | 499.97 | | -205.16 | | | 410.31 | | | 0.002 | | | 0.968 |
|  |  |  | |  | |  | | |  | | |  | | |  |
| **Pairwise differences (height*time)** | | | | | | | | | | | | | | | |
| emmeans(Full.mod, list(pairwise ~ height*time), adjust = tukey, type = response) | | | | | | | | | | | | | | | |
|  | | | | | | | | | | | | | | | |
| contrast | | | estimate | | SE | | | df | | t.ratio | | | | p.value | |
| LM,0 - EM,120 | | | 0.560 | | 0.089 | | | 93 | | 6.324 | | | | <0.001*** | |
| EA,0 - EM,120 | | | 0.515 | | 0.091 | | | 93 | | 5.643 | | | | <0.001*** | |
| LA,0 - EM,80 | | | 0.613 | | 0.087 | | | 93 | | 7.025 | | | | <0.001*** | |
| LA,0 - EM,120 | | | 0.655 | | 0.084 | | | 93 | | 7.825 | | | | <0.001*** | |
| EA,40 - EM,120 | | | 0.493 | | 0.091 | | | 93 | | 5.435 | | | | <0.001*** | |
| EA,80 - EM,120 | | | 0.531 | | 0.092 | | | 93 | | 5.773 | | | | <0.001*** | |
| LM,0 - EM,80 | | | 0.519 | | 0.090 | | | 93 | | 5.750 | | | | <0.001*** | |
| LM,40 - EM,120 | | | 0.466 | | 0.090 | | | 93 | | 5.184 | | | | <0.001*** | |
| LA,40 - EM,120 | | | 0.483 | | 0.092 | | | 93 | | 5.257 | | | | <0.001*** | |
| EM,80 - EA,80 | | | -0.489 | | 0.095 | | | 93 | | -5.130 | | | | <0.001*** | |
| EA,0 - EM,80 | | | 0.474 | | 0.095 | | | 93 | | 5.005 | | | | <0.001*** | |
| EA,40 - EM,80 | | | 0.452 | | 0.094 | | | 93 | | 4.797 | | | | <0.001*** | |
| LA,80 - EM,120 | | | 0.441 | | 0.091 | | | 93 | | 4.827 | | | | <0.001*** | |
| LA,40 - EM,80 | | | 0.441 | | 0.095 | | | 93 | | 4.658 | | | | 0.001** | |
| LM,40 - EM,80 | | | 0.424 | | 0.092 | | | 93 | | 4.611 | | | | 0.001** | |
| EM,120 - LA,120 | | | -0.398 | | 0.090 | | | 93 | | -4.442 | | | | 0.002** | |
| EM,0 - EM,120 | | | 0.423 | | 0.096 | | | 93 | | 4.413 | | | | 0.003** | |
| EM,120 - EA,120 | | | -0.402 | | 0.093 | | | 93 | | -4.327 | | | | 0.004** | |
| EM,80 - LA,80 | | | -0.399 | | 0.094 | | | 93 | | -4.257 | | | | 0.005** | |
| LA,0 - LM,120 | | | 0.422 | | 0.105 | | | 93 | | 4.039 | | | | 0.010* | |
| EM,0 - EM,80 | | | 0.381 | | 0.096 | | | 93 | | 3.962 | | | | 0.013* | |
| EM,80 - LA,120 | | | -0.356 | | 0.091 | | | 93 | | -3.899 | | | | 0.016* | |
| EM,80 - EA,120 | | | -0.361 | | 0.096 | | | 93 | | -3.752 | | | | 0.025* | |
| LM,80 - EM,120 | | | 0.326 | | 0.088 | | | 93 | | 3.701 | | | | 0.030* | |
| LA,0 - EM,40 | | | 0.399 | | 0.108 | | | 93 | | 3.684 | | | | 0.031* | |
| EM,40 - EM,120 | | | 0.255 | | 0.078 | | | 93 | | 3.263 | | | | 0.101 | |
| EM,80 - LM,80 | | | -0.285 | | 0.091 | | | 93 | | -3.134 | | | | 0.139 | |
| EM,120 - LM,120 | | | -0.233 | | 0.074 | | | 93 | | -3.126 | | | | 0.142 | |
| LM,0 - LM,120 | | | 0.328 | | 0.105 | | | 93 | | 3.120 | | | | 0.144 | |
| EA,0 - LM,120 | | | 0.283 | | 0.095 | | | 93 | | 2.978 | | | | 0.199 | |
| LA,0 - LM,80 | | | 0.328 | | 0.116 | | | 93 | | 2.841 | | | | 0.265 | |
| LM,0 - EM,40 | | | 0.305 | | 0.108 | | | 93 | | 2.820 | | | | 0.277 | |
| EA,80 - LM,120 | | | 0.298 | | 0.106 | | | 93 | | 2.811 | | | | 0.281 | |
| EM,40 - EM,80 | | | 0.214 | | 0.078 | | | 93 | | 2.755 | | | | 0.314 | |
| EA,40 - LM,120 | | | 0.261 | | 0.101 | | | 93 | | 2.591 | | | | 0.418 | |
| LA,0 - EA,120 | | | 0.253 | | 0.101 | | | 93 | | 2.508 | | | | 0.475 | |
| EM,80 - LM,120 | | | -0.191 | | 0.078 | | | 93 | | -2.437 | | | | 0.526 | |
| EM,40 - EA,80 | | | -0.275 | | 0.116 | | | 93 | | -2.377 | | | | 0.569 | |
| LA,0 - LA,120 | | | 0.257 | | 0.110 | | | 93 | | 2.342 | | | | 0.594 | |
| LM,40 - LM,120 | | | 0.233 | | 0.101 | | | 93 | | 2.309 | | | | 0.618 | |
| EA,0 - EM,40 | | | 0.260 | | 0.115 | | | 93 | | 2.251 | | | | 0.660 | |
| LA,40 - LM,120 | | | 0.250 | | 0.112 | | | 93 | | 2.241 | | | | 0.667 | |
| LM,0 - LM,80 | | | 0.234 | | 0.109 | | | 93 | | 2.143 | | | | 0.733 | |
| EM,40 - EA,40 | | | -0.238 | | 0.115 | | | 93 | | -2.068 | | | | 0.780 | |
| EM,40 - LA,40 | | | -0.227 | | 0.113 | | | 93 | | -2.020 | | | | 0.808 | |
| LA,0 - LA,80 | | | 0.214 | | 0.106 | | | 93 | | 2.016 | | | | 0.810 | |
| EM,0 - LA,0 | | | -0.232 | | 0.119 | | | 93 | | -1.955 | | | | 0.843 | |
| EM,40 - LM,40 | | | -0.210 | | 0.111 | | | 93 | | -1.894 | | | | 0.872 | |
| LA,80 - LM,120 | | | 0.208 | | 0.112 | | | 93 | | 1.858 | | | | 0.888 | |
| LA,0 - LA,40 | | | 0.172 | | 0.099 | | | 93 | | 1.735 | | | | 0.932 | |
| LM,80 - EA,80 | | | -0.204 | | 0.118 | | | 93 | | -1.735 | | | | 0.932 | |
| EA,0 - LM,80 | | | 0.189 | | 0.110 | | | 93 | | 1.720 | | | | 0.936 | |
| EM,40 - LA,80 | | | -0.185 | | 0.110 | | | 93 | | -1.688 | | | | 0.945 | |
| EM,0 - LM,120 | | | 0.190 | | 0.116 | | | 93 | | 1.635 | | | | 0.957 | |
| EM,0 - EM,40 | | | 0.167 | | 0.103 | | | 93 | | 1.626 | | | | 0.959 | |
| LA,0 - LM,40 | | | 0.189 | | 0.118 | | | 93 | | 1.609 | | | | 0.963 | |
| LM,120 - EA,120 | | | -0.170 | | 0.110 | | | 93 | | -1.547 | | | | 0.974 | |
| LM,120 - LA,120 | | | -0.165 | | 0.111 | | | 93 | | -1.485 | | | | 0.982 | |
| EA,40 - LM,80 | | | 0.167 | | 0.114 | | | 93 | | 1.469 | | | | 0.983 | |
| LA,0 - EA,40 | | | 0.161 | | 0.110 | | | 93 | | 1.467 | | | | 0.984 | |
| LM,40 - LM,80 | | | 0.139 | | 0.101 | | | 93 | | 1.376 | | | | 0.991 | |
| LM,0 - LA,120 | | | 0.163 | | 0.120 | | | 93 | | 1.360 | | | | 0.992 | |
| EM,40 - LA,120 | | | -0.142 | | 0.105 | | | 93 | | -1.359 | | | | 0.992 | |
| LA,40 - LM,80 | | | 0.157 | | 0.122 | | | 93 | | 1.286 | | | | 0.996 | |
| LM,0 - EA,120 | | | 0.158 | | 0.124 | | | 93 | | 1.281 | | | | 0.996 | |
| EM,40 - EA,120 | | | -0.147 | | 0.116 | | | 93 | | -1.266 | | | | 0.996 | |
| EA,80 - EA,120 | | | 0.129 | | 0.104 | | | 93 | | 1.234 | | | | 0.997 | |
| EA,0 - LA,0 | | | -0.139 | | 0.114 | | | 93 | | -1.228 | | | | 0.997 | |
| LA,0 - EA,80 | | | 0.124 | | 0.106 | | | 93 | | 1.166 | | | | 0.999 | |
| EM,0 - LM,0 | | | -0.137 | | 0.122 | | | 93 | | -1.125 | | | | 0.999 | |
| EM,80 - EM,120 | | | 0.042 | | 0.038 | | | 93 | | 1.107 | | | | 0.999 | |
| EA,80 - LA,120 | | | 0.133 | | 0.120 | | | 93 | | 1.108 | | | | 0.999 | |
| LM,80 - LM,120 | | | 0.094 | | 0.094 | | | 93 | | 1.002 | | | | 1.000 | |
| LM,0 - LA,80 | | | 0.119 | | 0.122 | | | 93 | | 0.979 | | | | 1.000 | |
| EA,0 - EA,120 | | | 0.113 | | 0.117 | | | 93 | | 0.970 | | | | 1.000 | |
| EA,0 - LA,120 | | | 0.118 | | 0.122 | | | 93 | | 0.964 | | | | 1.000 | |
| LM,0 - LM,40 | | | 0.095 | | 0.102 | | | 93 | | 0.933 | | | | 1.000 | |
| LM,80 - LA,80 | | | -0.115 | | 0.122 | | | 93 | | -0.941 | | | | 1.000 | |
| EM,0 - EA,0 | | | -0.092 | | 0.127 | | | 93 | | -0.726 | | | | 1.000 | |
| EM,0 - LM,40 | | | -0.043 | | 0.125 | | | 93 | | -0.344 | | | | 1.000 | |
| EM,0 - EA,40 | | | -0.071 | | 0.127 | | | 93 | | -0.558 | | | | 1.000 | |
| EM,0 - LA,40 | | | -0.060 | | 0.121 | | | 93 | | -0.497 | | | | 1.000 | |
| EM,0 - LM,80 | | | 0.096 | | 0.125 | | | 93 | | 0.774 | | | | 1.000 | |
| EM,0 - EA,80 | | | -0.108 | | 0.127 | | | 93 | | -0.852 | | | | 1.000 | |
| EM,0 - LA,80 | | | -0.018 | | 0.117 | | | 93 | | -0.155 | | | | 1.000 | |
| EM,0 - EA,120 | | | 0.021 | | 0.126 | | | 93 | | 0.164 | | | | 1.000 | |
| EM,0 - LA,120 | | | 0.025 | | 0.109 | | | 93 | | 0.232 | | | | 1.000 | |
| LM,0 - EA,0 | | | 0.045 | | 0.118 | | | 93 | | 0.383 | | | | 1.000 | |
| LM,0 - LA,0 | | | -0.094 | | 0.117 | | | 93 | | -0.804 | | | | 1.000 | |
| LM,0 - EA,40 | | | 0.067 | | 0.120 | | | 93 | | 0.559 | | | | 1.000 | |
| LM,0 - LA,40 | | | 0.077 | | 0.123 | | | 93 | | 0.630 | | | | 1.000 | |
| LM,0 - EA,80 | | | 0.030 | | 0.122 | | | 93 | | 0.242 | | | | 1.000 | |
| EA,0 - LM,40 | | | 0.050 | | 0.115 | | | 93 | | 0.431 | | | | 1.000 | |
| EA,0 - EA,40 | | | 0.022 | | 0.101 | | | 93 | | 0.215 | | | | 1.000 | |
| EA,0 - LA,40 | | | 0.032 | | 0.121 | | | 93 | | 0.266 | | | | 1.000 | |
| EA,0 - EA,80 | | | -0.016 | | 0.110 | | | 93 | | -0.141 | | | | 1.000 | |
| EA,0 - LA,80 | | | 0.074 | | 0.122 | | | 93 | | 0.608 | | | | 1.000 | |
| EM,40 - LM,80 | | | -0.071 | | 0.111 | | | 93 | | -0.637 | | | | 1.000 | |
| EM,40 - LM,120 | | | 0.023 | | 0.103 | | | 93 | | 0.223 | | | | 1.000 | |
| LM,40 - EA,40 | | | -0.028 | | 0.118 | | | 93 | | -0.236 | | | | 1.000 | |
| LM,40 - LA,40 | | | -0.017 | | 0.123 | | | 93 | | -0.140 | | | | 1.000 | |
| LM,40 - EA,80 | | | -0.065 | | 0.121 | | | 93 | | -0.539 | | | | 1.000 | |
| LM,40 - LA,80 | | | 0.025 | | 0.123 | | | 93 | | 0.202 | | | | 1.000 | |
| LM,40 - EA,120 | | | 0.064 | | 0.123 | | | 93 | | 0.517 | | | | 1.000 | |
| LM,40 - LA,120 | | | 0.068 | | 0.121 | | | 93 | | 0.562 | | | | 1.000 | |
| EA,40 - LA,40 | | | 0.010 | | 0.118 | | | 93 | | 0.088 | | | | 1.000 | |
| EA,40 - EA,80 | | | -0.037 | | 0.102 | | | 93 | | -0.367 | | | | 1.000 | |
| EA,40 - LA,80 | | | 0.053 | | 0.120 | | | 93 | | 0.437 | | | | 1.000 | |
| EA,40 - EA,120 | | | 0.091 | | 0.111 | | | 93 | | 0.824 | | | | 1.000 | |
| EA,40 - LA,120 | | | 0.096 | | 0.121 | | | 93 | | 0.794 | | | | 1.000 | |
| LA,40 - EA,80 | | | -0.048 | | 0.116 | | | 93 | | -0.413 | | | | 1.000 | |
| LA,40 - LA,80 | | | 0.042 | | 0.103 | | | 93 | | 0.410 | | | | 1.000 | |
| LA,40 - EA,120 | | | 0.081 | | 0.112 | | | 93 | | 0.723 | | | | 1.000 | |
| LA,40 - LA,120 | | | 0.085 | | 0.110 | | | 93 | | 0.778 | | | | 1.000 | |
| LM,80 - EA,120 | | | -0.076 | | 0.120 | | | 93 | | -0.630 | | | | 1.000 | |
| LM,80 - LA,120 | | | -0.071 | | 0.120 | | | 93 | | -0.592 | | | | 1.000 | |
| EA,80 - LA,80 | | | 0.090 | | 0.119 | | | 93 | | 0.756 | | | | 1.000 | |
| LA,80 - EA,120 | | | 0.039 | | 0.117 | | | 93 | | 0.333 | | | | 1.000 | |
| LA,80 - LA,120 | | | 0.043 | | 0.102 | | | 93 | | 0.425 | | | | 1.000 | |
| EA,120 - LA,120 | | | 0.004 | | 0.119 | | | 93 | | 0.038 | | | | 1.000 | |

**Table C. Model 3 - Juvenile count**

| **Effect of interaction between time and height** | | | | | | | | | | | | | |
| --- | --- | --- | --- | --- | --- | --- | --- | --- | --- | --- | --- | --- | --- |
| Analysis of Variance Table using likelihood ratio tests | | | | | | | | | | | | | |
| Full.mod.int = glmmTMB(Juvenile ~ height * time + (1\|date), | | | | | | | | | | | | | |
| ziformula=~1, family=poisson) | | | | | | | | | | | | | |
| Full.mod = glmmtTMB(Juvenile ~ height + time + (1\|date), | | | | | | | | | | | | | |
| ziformula=~1, family=poisson) | | | | | | | | | | | | | |
|  | Df | | AIC | | BIC | logLik | | | Dev | Chisq | | Pr(>Chisq) | |
| Full.mod | 9 | | 139.80 | | 164.26 | -60.899 | | | 121.80 |  | |  | |
| Full.mod.int | 18 | | 149.80 | | 198.74 | -56.902 | | | 113.80 | 7.994 | | 0.535 | |
|  |  | |  | |  |  | | |  |  | |  | |
| **Effect of height** | | | | | | | | | | | | | |
| Analysis of Variance Table using likelihood ratio tests | | | | | | | | | | | | | |
| Full.mod = glmmTMB(Juvenile ~ height + time + (1\|date), | | | | | | | | | | | | | |
| ziformula=~1, family=poisson) | | | | | | | | | | | | | |
| Red.mod = glmmtTMB(Juvenile ~ time + (1\|date), | | | | | | | | | | | | | |
| ziformula=~1, family=poisson) | | | | | | | | | | | | | |
|  | Df | | AIC | | BIC | logLik | | | Dev | Chisq | | Pr(>Chisq) | |
| Red.mod | 6 | | 152.97 | | 169.28 | -70.485 | | | 140.97 |  | |  | |
| Full.mod | 9 | | 139.80 | | 164.26 | -60.899 | | | 121.80 | 19.172 | | <0.001*** | |
|  |  | |  | |  |  | | |  |  | |  | |
| **Effect of time** | | | | | | | | | | | | | |
| Analysis of Variance Table using likelihood ratio tests | | | | | | | | | | | | | |
| Full.mod = glmmTMB(Juvenile ~ height + time + (1\|date), | | | | | | | | | | | | | |
| ziformula=~1, family=poisson) | | | | | | | | | | | | | |
| Red.mod = glmmtTMB(Juvenile ~ height + (1\|date), | | | | | | | | | | | | | |
| ziformula=~1, family=poisson) | | | | | | | | | | | | | |
|  | Df | | AIC | | BIC | logLik | | | Dev | Chisq | | Pr(>Chisq) | |
| Red.mod | 6 | | 136.96 | | 153.27 | -62.478 | | | 124.96 |  | |  | |
| Full.mod | 9 | | 139.80 | | 164.26 | -60.899 | | | 121.80 | 3.158 | | 0.368 | |
|  |  | |  | |  |  | | |  |  | |  | |
| **Effect of date (random effect)** | | | | | | | | | | | | | |
| Analysis of Variance Table using likelihood ratio tests | | | | | | | | | | | | | |
| Full.mod = glmmTMB(Juvenile ~ height + time + (1\|date), | | | | | | | | | | | | | |
| ziformula=~1, family=poisson) | | | | | | | | | | | | | |
| Red.mod = glmmtTMB(Juvenile ~ height + time, | | | | | | | | | | | | | |
| ziformula=~1, family=poisson) | | | | | | | | | | | | | |
|  | Df | | AIC | | BIC | logLik | | | Dev | Chisq | | Pr(>Chisq) | |
| Red.mod | 8 | | 137.85 | | 159.59 | -60.923 | | | 121.85 |  | |  | |
| Full.mod | 9 | | 139.80 | | 164.26 | -60.899 | | | 121.80 | 0.048 | | 0.826 | |
|  |  | |  | |  |  | | |  |  | |  | |
| **Emmeans and pairwise differences (height)** | | | | | | | | | | | | | |
| emmeans(Full.mod, list(pairwise ~ height), adjust = tukey) | | | | | | | | | | | | | |
| height | | emmean | | SE | | | df | lower.CL | | | upper.CL | |  |
| Land count | | 0.63 | | 0.162 | | | 103 | 0.38 | | | 1.05 | |  |
| 40 | | 0.20 | | 0.085 | | | 103 | 0.09 | | | 0.46 | |  |
| 80 | | 0.17 | | 0.077 | | | 103 | 0.07 | | | 0.42 | |  |
| 120 | | 0.07 | | 0.048 | | | 103 | 0.02 | | | 0.28 | |  |
|  | |  | |  | | |  |  | | |  | |  |
| contrast | | | | estimate | | | SE | df | | | t.ratio | | p.value |
| Land count - 40 | | | | 1.153 | | | 0.468 | 103 | | | 2.461 | | 0.072. |
| Land count - 80 | | | | 1.335 | | | 0.503 | 103 | | | 2.656 | | 0.045* |
| Land count - 120 | | | | 2.251 | | | 0.743 | 103 | | | 3.028 | | 0.016* |
| 40 - 80 | | | | 0.182 | | | 0.606 | 103 | | | 0.301 | | 0.991 |
| 40 – 120 | | | | 1.099 | | | 0.816 | 103 | | | 1.346 | | 0.536 |
| 80 - 120 | | | | 0.916 | | | 0.837 | 103 | | | 1.095 | | 0.693 |
| Results are averaged over the levels of: time  Degrees-of-freedom method: kenward-roger  Confidence level used: 0.95  P value adjustment: tukey method for comparing family of 4 estimates | | | | | | | | | | | | | |
| Results are averaged over the levels of: time  Confidence level used: 0.95  P value adjustment: tukey method for comparing family of 4 estimates | | | | | | | | | | | | | |

**Table D. Model 4 - Subadult count**

| **Effect of interaction between time and height** | | | | | | | | | | | | | |
| --- | --- | --- | --- | --- | --- | --- | --- | --- | --- | --- | --- | --- | --- |
| Analysis of Variance Table using likelihood ratio tests | | | | | | | | | | | | | |
| Full.mod.int = glmmTMB(Subadult ~ height * time + (1\|date), | | | | | | | | | | | | | |
| ziformula=~1, family=poisson) | | | | | | | | | | | | | |
| Full.mod = glmmtTMB(Subadult ~ height + time + (1\|date), | | | | | | | | | | | | | |
| ziformula=~1, family=poisson) | | | | | | | | | | | | | |
|  | Df | | AIC | | BIC | logLik | | | Dev | Chisq | | Pr(>Chisq) | |
| Full.mod | 9 | | 152.44 | | 176.90 | -67.219 | | | 134.44 |  | |  | |
| Full.mod.int | 18 | | 157.80 | | 206.73 | -60.899 | | | 121.80 | 12.640 | | 0.180 | |
|  |  | |  | |  |  | | |  |  | |  | |
| **Effect of height** | | | | | | | | | | | | | |
| Analysis of Variance Table using likelihood ratio tests | | | | | | | | | | | | | |
| Full.mod = glmmTMB(Subadult ~ height + time + (1\|date), | | | | | | | | | | | | | |
| ziformula=~1, family=poisson) | | | | | | | | | | | | | |
| Red.mod = glmmtTMB(Subadult ~ time + (1\|date), | | | | | | | | | | | | | |
| ziformula=~1, family=poisson) | | | | | | | | | | | | | |
|  | Df | | AIC | | BIC | logLik | | | Dev | Chisq | | Pr(>Chisq) | |
| Red.mod | 6 | | 170.59 | | 186.90 | -79.294 | | | 158.59 |  | |  | |
| Full.mod | 9 | | 152.44 | | 176.90 | -67.219 | | | 134.44 | 24.151 | | <0.001*** | |
|  |  | |  | |  |  | | |  |  | |  | |
| **Effect of time** | | | | | | | | | | | | | |
| Analysis of Variance Table using likelihood ratio tests | | | | | | | | | | | | | |
| Full.mod = glmmTMB(Subadult ~ height + time + (1\|date), | | | | | | | | | | | | | |
| ziformula=~1, family=poisson) | | | | | | | | | | | | | |
| Red.mod = glmmtTMB(Subadult ~ height + (1\|date), | | | | | | | | | | | | | |
| ziformula=~1, family=poisson) | | | | | | | | | | | | | |
|  | Df | | AIC | | BIC | logLik | | | Dev | Chisq | | Pr(>Chisq) | |
| Red.mod | 6 | | 157.33 | | 173.65 | -72.667 | | | 145.33 |  | |  | |
| Full.mod | 9 | | 152.44 | | 176.90 | -67.219 | | | 134.44 | 10.896 | | 0.012* | |
|  |  | |  | |  |  | | |  |  | |  | |
| **Effect of date (random effect)** | | | | | | | | | | | | | |
| Analysis of Variance Table using likelihood ratio tests | | | | | | | | | | | | | |
| Full.mod = glmmTMB(Subadult ~ height + time + (1\|date), | | | | | | | | | | | | | |
| ziformula=~1, family=poisson) | | | | | | | | | | | | | |
| Red.mod = glmmtTMB(Subadult ~ height + time, | | | | | | | | | | | | | |
| ziformula=~1, family=poisson) | | | | | | | | | | | | | |
|  | Df | | AIC | | BIC | logLik | | | Dev | Chisq | | Pr(>Chisq) | |
| Red.mod | 8 | | 150.94 | | 172.69 | -67.471 | | | 134.94 |  | |  | |
| Full.mod | 9 | | 152.44 | | 176.90 | -67.219 | | | 134.44 | 0.505 | | 0.477 | |
|  |  | |  | |  |  | | |  |  | |  | |
| **Emmeans and pairwise differences (height)** | | | | | | | | | | | | | |
| emmeans(Full.mod, list(pairwise ~ height), adjust = tukey) | | | | | | | | | | | | | |
| height | | emmean | | SE | | | df | lower.CL | | | upper.CL | |  |
| Land count | | 0.67 | | 0.177 | | | 103 | 0.39 | | | 1.13 | |  |
| 40 | | 0.17 | | 0.077 | | | 103 | 0.07 | | | 0.42 | |  |
| 80 | | 0.23 | | 0.091 | | | 103 | 0.11 | | | 0.50 | |  |
| 120 | | 0.06 | | 0.042 | | | 103 | 0.01 | | | 0.25 | |  |
|  | |  | |  | | |  |  | | |  | |  |
| contrast | | | | estimate | | | SE | df | | | t.ratio | | p.value |
| Land count - 40 | | | | 1.344 | | | 0.458 | 103 | | | 2.931 | | 0.021* |
| Land count - 80 | | | | 1.056 | | | 0.410 | 103 | | | 2.573 | | 0.055. |
| Land count - 120 | | | | 2.442 | | | 0.737 | 103 | | | 3.313 | | 0.007** |
| 40 - 80 | | | | -0.288 | | | 0.540 | 103 | | | -0.533 | | 0.951 |
| 40 – 120 | | | | 1.099 | | | 0.816 | 103 | | | 1.346 | | 0.536 |
| 80 - 120 | | | | 1.386 | | | 0.791 | 103 | | | 1.754 | | 0.302 |
| Results are averaged over the levels of: time  Degrees-of-freedom method: kenward-roger  Confidence level used: 0.95  P value adjustment: tukey method for comparing family of 4 estimates | | | | | | | | | | | | | |
| Results are averaged over the levels of: time  Confidence level used: 0.95  P value adjustment: tukey method for comparing family of 4 estimates | | | | | | | | | | | | | |
| **Emmeans and pairwise differences (time)** | | | | | | | | | | | | | |
| emmeans(Full.mod, list(pairwise ~ time), adjust = tukey) | | | | | | | | | | | | | |
| time | | emmean | | SE | | | df | lower.CL | | | upper.CL | |  |
| EM | | 0.07 | | 0.044 | | | 103 | 0.02 | | | 0.24 | |  |
| LM | | 0.22 | | 0.085 | | | 103 | 0.10 | | | 0.47 | |  |
| EA | | 0.24 | | 0.091 | | | 103 | 0.11 | | | 0.51 | |  |
| LA | | 0.41 | | 0.130 | | | 103 | 0.22 | | | 0.77 | |  |
|  | |  | |  | | |  |  | | |  | |  |
| contrast | | | | contrast | | | SE | df | | | t.ratio | | p.value |
| EM – LM | | | | -1.099 | | | 0.667 | 103 | | | -1.648 | | 0.357 |
| EM – EA | | | | -1.204 | | | 0.658 | 103 | | | -1.829 | | 0.266 |
| EM – LA | | | | -1.735 | | | 0.626 | 103 | | | -2.770 | | 0.033* |
| LM – EA | | | | -0.105 | | | 0.459 | 103 | | | -0.229 | | 0.996 |
| LM – LA | | | | -0.636 | | | 0.412 | 103 | | | -1.543 | | 0.416 |
| EA - LA | | | | -0.531 | | | 0.399 | 103 | | | -1.331 | | 0.545 |
| Results are averaged over the levels of: time  Degrees-of-freedom method: kenward-roger  Confidence level used: 0.95  P value adjustment: tukey method for comparing family of 4 estimates | | | | | | | | | | | | | |
| Results are averaged over the levels of: height  Confidence level used: 0.95  P value adjustment: tukey method for comparing family of 4 estimates | | | | | | | | | | | | | |

**Table E. Model 5 - Adult count**

| **Effect of inclusion of AR(1) variable** | | | | | | | | | | | | | | | |
| --- | --- | --- | --- | --- | --- | --- | --- | --- | --- | --- | --- | --- | --- | --- | --- |
| Analysis of Variance Table using likelihood ratio tests | | | | | | | | | | | | | | | |
| Mod.AR1 = glmmTMB(Adult ~ height*time + (1\|date)  xfchbxfdghfgh  ) | | | | | | | | | | | | | | | |
| + ar1(times+0\|group), ziformula=~1, family=poisson)  weights=total) | | | | | | | | | | | | | | | |
| Mod = glmmTMB(Adult ~ height*time + (1\|date), | | | | | | | | | | | | | | | |
| ziformula=~1, family=poisson) | | | | | | | | | | | | | | | |
|  | Df | AIC | | BIC | | logLik | | | Dev | | | Chisq | | | Pr(>Chisq) |
| Mod | 18 | 456.49 | | 505.42 | | -210.25 | | | 420.49 | | |  | | |  |
| Mod.AR1 | 20 | 454.26 | | 508.63 | | -207.13 | | | 414.26 | | | 6.228 | | | 0.044* |
|  |  |  | |  | |  | | |  | | |  | | |  |
| **Effect of interaction between time and height** | | | | | | | | | | | | | | | |
| Analysis of Variance Table using likelihood ratio tests | | | | | | | | | | | | | | | |
| Full.mod = glmmTMB(Adult ~ height * time + (1\|date) | | | | | | | | | | | | | | | |
| + ar1(times+0\|group), ziformula=~1, family=poisson) | | | | | | | | | | | | | | | |
| Full.mod.add = glmmTMB(Adult ~ height + time + (1\|date) | | | | | | | | | | | | | | | |
| + ar1(times+0\|group), ziformula=~1, family=poisson) | | | | | | | | | | | | | | | |
|  | | Df | AIC | | BIC | | logLik | | | | Dev | | Chisq | | Pr(>Chisq) |
| Full.mod.adsd | | 11 | 461.12 | | 491.02 | | -219.56 | | | | 439.12 | |  | |  |
| Full.mod | | 20 | 454.26 | | 508.63 | | -207.13 | | | | 414.26 | | 24.854 | | 0.003** |
|  | |  |  | |  | |  | | | |  | |  | |  |
| **Effect of date (random effect)** | | | | | | | | | | | | | | | |
| Analysis of Variance Table using likelihood ratio tests | | | | | | | | | | | | | | | |
| Full.mod = glmmTMB(Adult ~ height * time + (1\|date)  + ar1(times+0\|group), ziformula=~1, family=poisson) | | | | | | | | | | | | | | | |
| Red.mod = glmmTMB(Adult ~ height * time + | | | | | | | | | | | | | | | |
| + ar1(times+0\|group), ziformula=~1, family=poisson) | | | | | | | | | | | | | | | |
|  | Df | AIC | | BIC | | logLik | | | Dev | | | Chisq | | | Pr(>Chisq) |
| Red.mod | 19 | 452.26 | | 503.92 | | -207.13 | | | 414.26 | | |  | | |  |
| Full.mo | 20 | 454.26 | | 508.63 | | -207.13 | | | 414.26 | | | 0 | | | 1.000 |
|  |  |  | |  | |  | | |  | | |  | | |  |
| **Pairwise differences (height*time)** | | | | | | | | | | | | | | | |
| emmeans(Full.mod, list(pairwise ~ height*time), adjust = tukey, type = response) | | | | | | | | | | | | | | | |
|  | | | | | | | | | | | | | | | |
| contrast | | | estimate | | SE | | | df | | t.ratio | | | | p.value | |

| EA,40 - EM,120 | 4.798 | 1.064 | 94 | 4.510 | 0.002** |
| --- | --- | --- | --- | --- | --- |
| EA,80 - EM,120 | 4.531 | 1.023 | 94 | 4.428 | 0.003** |
| LM,40 - EM,120 | 4.398 | 1.003 | 94 | 4.385 | 0.003** |
| LM,0 - EM,120 | 3.998 | 0.941 | 94 | 4.248 | 0.005** |
| LA,40 - EM,120 | 3.865 | 0.921 | 94 | 4.198 | 0.006** |
| EA,40 - EM,80 | 4.388 | 1.056 | 94 | 4.157 | 0.007** |
| EA,0 - EM,120 | 3.731 | 0.900 | 94 | 4.147 | 0.007** |
| LA,80 - EM,120 | 3.731 | 0.900 | 94 | 4.147 | 0.007** |
| EM,120 - LA,120 | -3.598 | 0.879 | 94 | -4.094 | 0.008** |
| EM,120 - EA,120 | -3.801 | 0.933 | 94 | -4.074 | 0.009** |
| EM,80 - EA,80 | -4.122 | 1.017 | 94 | -4.053 | 0.010* |
| LA,0 - EM,120 | 3.465 | 0.858 | 94 | 4.038 | 0.010* |
| LM,40 - EM,80 | 3.988 | 0.997 | 94 | 3.999 | 0.011* |
| LM,0 - EM,80 | 3.588 | 0.939 | 94 | 3.822 | 0.020* |
| LA,40 - EM,80 | 3.455 | 0.919 | 94 | 3.758 | 0.025* |
| EA,0 - EM,80 | 3.322 | 0.900 | 94 | 3.692 | 0.030* |
| EM,80 - LA,80 | -3.322 | 0.900 | 94 | -3.692 | 0.030* |
| EM,80 - EA,120 | -3.391 | 0.931 | 94 | -3.642 | 0.035* |
| EM,80 - LA,120 | -3.188 | 0.880 | 94 | -3.623 | 0.037* |
| LA,0 - EM,80 | 3.055 | 0.860 | 94 | 3.551 | 0.046* |
| LM,80 - EM,120 | 2.748 | 0.775 | 94 | 3.545 | 0.047* |
| EM,120 - LM,120 | -2.265 | 0.663 | 94 | -3.414 | 0.067. |
| EM,0 - EM,120 | 2.044 | 0.643 | 94 | 3.177 | 0.125 |
| EM,80 - LM,80 | -2.339 | 0.784 | 94 | -2.985 | 0.196 |
| EM,40 - EM,120 | 3.158 | 1.150 | 94 | 2.747 | 0.318 |
| EM,80 - LM,120 | -1.855 | 0.681 | 94 | -2.724 | 0.332 |
| EM,0 - EA,40 | -2.754 | 1.058 | 94 | -2.602 | 0.410 |
| EM,0 - EM,80 | 1.634 | 0.663 | 94 | 2.464 | 0.506 |
| EM,0 - EA,80 | -2.487 | 1.028 | 94 | -2.419 | 0.539 |
| EA,40 - LM,120 | 2.534 | 1.051 | 94 | 2.410 | 0.545 |
| EM,40 - EM,80 | 2.749 | 1.149 | 94 | 2.392 | 0.558 |
| EM,0 - LM,40 | -2.354 | 1.013 | 94 | -2.323 | 0.608 |
| EA,80 - LM,120 | 2.267 | 1.022 | 94 | 2.218 | 0.682 |
| LM,40 - LM,120 | 2.134 | 1.007 | 94 | 2.118 | 0.749 |
| EM,0 - LM,0 | -1.954 | 0.969 | 94 | -2.016 | 0.810 |
| EM,0 - LA,40 | -1.821 | 0.955 | 94 | -1.907 | 0.866 |
| EA,40 - LM,80 | 2.050 | 1.082 | 94 | 1.895 | 0.872 |
| EM,0 - EA,120 | -1.757 | 0.964 | 94 | -1.822 | 0.902 |
| LM,0 - LM,120 | 1.733 | 0.965 | 94 | 1.797 | 0.911 |
| EM,0 - EA,0 | -1.687 | 0.940 | 94 | -1.794 | 0.912 |
| EM,0 - LA,80 | -1.687 | 0.940 | 94 | -1.794 | 0.912 |
| LM,80 - EA,80 | -1.783 | 1.056 | 94 | -1.689 | 0.945 |
| LA,40 - LM,120 | 1.600 | 0.951 | 94 | 1.683 | 0.946 |
| EM,0 - LA,120 | -1.554 | 0.926 | 94 | -1.677 | 0.948 |
| LM,120 - EA,120 | -1.536 | 0.965 | 94 | -1.592 | 0.966 |
| LM,40 - LM,80 | 1.650 | 1.043 | 94 | 1.582 | 0.968 |
| EA,0 - LM,120 | 1.467 | 0.937 | 94 | 1.565 | 0.971 |
| LA,80 - LM,120 | 1.467 | 0.937 | 94 | 1.565 | 0.971 |
| EM,0 - LA,0 | -1.421 | 0.912 | 94 | -1.557 | 0.972 |
| LM,120 - LA,120 | -1.333 | 0.924 | 94 | -1.444 | 0.986 |
| LA,0 - LM,120 | 1.200 | 0.910 | 94 | 1.318 | 0.994 |
| EM,80 - EM,120 | 0.410 | 0.312 | 94 | 1.313 | 0.995 |
| LM,0 - LM,80 | 1.250 | 1.006 | 94 | 1.242 | 0.997 |
| LA,0 - EA,40 | -1.333 | 1.083 | 94 | -1.231 | 0.997 |
| EM,40 - EA,40 | -1.640 | 1.353 | 94 | -1.211 | 0.998 |
| LA,40 - LM,80 | 1.116 | 0.994 | 94 | 1.123 | 0.999 |
| EA,40 - LA,120 | 1.200 | 1.088 | 94 | 1.103 | 0.999 |
| LM,80 - EA,120 | -1.052 | 0.999 | 94 | -1.053 | 1.000 |
| EM,40 - EA,80 | -1.373 | 1.334 | 94 | -1.029 | 1.000 |
| EA,0 - LM,80 | 0.983 | 0.982 | 94 | 1.001 | 1.000 |
| LA,0 - EA,80 | -1.067 | 1.061 | 94 | -1.006 | 1.000 |
| LM,80 - LA,80 | -0.983 | 0.982 | 94 | -1.001 | 1.000 |
| EA,0 - EA,40 | -1.067 | 1.094 | 94 | -0.975 | 1.000 |
| EA,40 - LA,80 | 1.067 | 1.094 | 94 | 0.975 | 1.000 |
| EM,0 - EM,40 | -1.114 | 1.184 | 94 | -0.941 | 1.000 |
| LA,0 - LM,40 | -0.933 | 1.050 | 94 | -0.889 | 1.000 |
| EM,40 - LM,40 | -1.240 | 1.325 | 94 | -0.936 | 1.000 |
| EA,40 - EA,120 | 0.997 | 1.113 | 94 | 0.896 | 1.000 |
| EM,0 - LM,80 | -0.704 | 0.863 | 94 | -0.817 | 1.000 |
| EM,0 - LM,120 | -0.220 | 0.796 | 94 | -0.277 | 1.000 |
| LM,0 - EA,0 | 0.267 | 1.034 | 94 | 0.258 | 1.000 |
| LM,0 - LA,0 | 0.533 | 1.018 | 94 | 0.524 | 1.000 |
| LM,0 - EM,40 | 0.840 | 1.298 | 94 | 0.647 | 1.000 |
| LM,0 - LM,40 | -0.400 | 1.077 | 94 | -0.372 | 1.000 |
| LM,0 - EA,40 | -0.800 | 1.105 | 94 | -0.724 | 1.000 |
| LM,0 - LA,40 | 0.133 | 1.042 | 94 | 0.128 | 1.000 |
| LM,0 - EA,80 | -0.533 | 1.086 | 94 | -0.491 | 1.000 |
| LM,0 - LA,80 | 0.267 | 1.034 | 94 | 0.258 | 1.000 |
| LM,0 - EA,120 | 0.197 | 1.056 | 94 | 0.187 | 1.000 |
| LM,0 - LA,120 | 0.400 | 1.026 | 94 | 0.390 | 1.000 |
| EA,0 - LA,0 | 0.267 | 0.999 | 94 | 0.267 | 1.000 |
| EA,0 - EM,40 | 0.573 | 1.281 | 94 | 0.447 | 1.000 |
| EA,0 - LM,40 | -0.667 | 1.063 | 94 | -0.627 | 1.000 |
| EA,0 - LA,40 | -0.133 | 1.024 | 94 | -0.130 | 1.000 |
| EA,0 - EA,80 | -0.800 | 1.073 | 94 | -0.746 | 1.000 |
| EA,0 - LA,80 | 0.000 | 1.016 | 94 | 0.000 | 1.000 |
| EA,0 - EA,120 | -0.069 | 1.038 | 94 | -0.067 | 1.000 |
| EA,0 - LA,120 | 0.133 | 1.007 | 94 | 0.132 | 1.000 |
| LA,0 - EM,40 | 0.306 | 1.265 | 94 | 0.242 | 1.000 |
| LA,0 - LA,40 | -0.400 | 1.008 | 94 | -0.397 | 1.000 |
| LA,0 - LM,80 | 0.716 | 0.960 | 94 | 0.747 | 1.000 |
| LA,0 - LA,80 | -0.267 | 0.999 | 94 | -0.267 | 1.000 |
| LA,0 - EA,120 | -0.336 | 1.022 | 94 | -0.329 | 1.000 |
| LA,0 - LA,120 | -0.133 | 0.989 | 94 | -0.135 | 1.000 |
| EM,40 - LA,40 | -0.706 | 1.289 | 94 | -0.548 | 1.000 |
| EM,40 - LM,80 | 0.410 | 1.203 | 94 | 0.341 | 1.000 |
| EM,40 - LA,80 | -0.573 | 1.281 | 94 | -0.447 | 1.000 |
| EM,40 - LM,120 | 0.894 | 1.204 | 94 | 0.742 | 1.000 |
| EM,40 - EA,120 | -0.642 | 1.270 | 94 | -0.506 | 1.000 |
| EM,40 - LA,120 | -0.439 | 1.273 | 94 | -0.345 | 1.000 |
| LM,40 - EA,40 | -0.400 | 1.125 | 94 | -0.356 | 1.000 |
| LM,40 - LA,40 | 0.533 | 1.069 | 94 | 0.499 | 1.000 |
| LM,40 - EA,80 | -0.133 | 1.108 | 94 | -0.120 | 1.000 |
| LM,40 - LA,80 | 0.667 | 1.063 | 94 | 0.628 | 1.000 |
| LM,40 - EA,120 | 0.597 | 1.083 | 94 | 0.551 | 1.000 |
| LM,40 - LA,120 | 0.800 | 1.056 | 94 | 0.758 | 1.000 |
| EA,40 - LA,40 | 0.933 | 1.099 | 94 | 0.849 | 1.000 |
| EA,40 - EA,80 | 0.267 | 1.132 | 94 | 0.236 | 1.000 |
| LA,40 - EA,80 | -0.667 | 1.079 | 94 | -0.618 | 1.000 |
| LA,40 - LA,80 | 0.133 | 1.024 | 94 | 0.130 | 1.000 |
| LA,40 - EA,120 | 0.064 | 1.047 | 94 | 0.061 | 1.000 |
| LA,40 - LA,120 | 0.267 | 1.016 | 94 | 0.262 | 1.000 |
| LM,80 - LM,120 | 0.484 | 0.870 | 94 | 0.556 | 1.000 |
| LM,80 - LA,120 | -0.850 | 0.971 | 94 | -0.875 | 1.000 |
| EA,80 - LA,80 | 0.800 | 1.073 | 94 | 0.746 | 1.000 |
| EA,80 - EA,120 | 0.731 | 1.093 | 94 | 0.669 | 1.000 |
| EA,80 - LA,120 | 0.933 | 1.066 | 94 | 0.875 | 1.000 |
| LA,80 - EA,120 | -0.069 | 1.038 | 94 | -0.067 | 1.000 |
| LA,80 - LA,120 | 0.133 | 1.007 | 94 | 0.132 | 1.000 |
| EA,120 - LA,120 | 0.203 | 1.030 | 94 | 0.197 | 1.000 |
