# Supplementary figures and images for: Drone-based effective counting and ageing of hippopotamus (Hippopotamus amphibius) in the Okavango Delta in Botswana
Source: PLoS One. 2019 Dec 5;14(12):e0219652. doi: 10.1371/journal.pone.0219652 (PMC6894862; doi:10.1371/journal.pone.0219652)

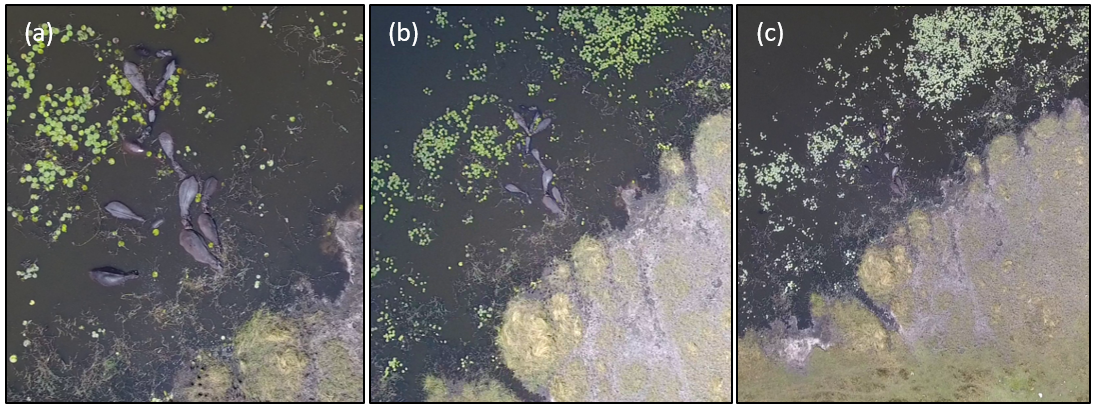

Supplement: S1 Fig — Snapshots of survey videos at a) 40 metres, b) 80 meters, c) 120 metres. Notice the increasing difficulty of detecting hippos with increasing altitude due to lowering resolution. Images taken during early afternoon surveys showing resting posture of hippos with the majority of their body exposed, allowing easy detection and counting. (PNG) [file pone.0219652.s003.png]
