# Supplementary material for: Drone-based effective counting and ageing of hippopotamus (Hippopotamus amphibius) in the Okavango Delta in Botswana
Source: PLoS One. 2019 Dec 5;14(12):e0219652. doi: 10.1371/journal.pone.0219652 (PMC6894862; doi:10.1371/journal.pone.0219652)
Supplement: Attachment — Submitted filename: Response to Reviewers.pdf [file pone.0219652.s005.pdf]

Response to reviewers

We have listed the Editor and reviewer comments below, with our responses in italics, including how we have changed the manuscript in inverted commas (with additional text in bold and removed original text crossed out).

Editor comments

1. The main issues relate to the statistical analyses. Reviewer #2 provides some very specific suggestions concerning the models that should be used given the data you have on hand. Please address this issue with an appropriate re-analysis. Reviewer #2 also suggests that the degrees of freedom should be adjusted for possible bias due to small sample sizes. This should also be addressed.

   Response: *We have addressed reviewer 2's suggestions below.*

2. Finally, is the "true" number of hippos actually known? If so, this should be used to assess the relative performance of the drone and land-based survey methods. I would imagine that for such a species, this might actually be possible!

   Response: *Unfortunately, the true number of hippos in the lagoon on the days of the survey could not be determined, even with small numbers, because of their behaviour (lines 64-65) (Klingel, 2013; Delvingt, 1978). This is because hippos in the water continually surface and submerge and individuals are not easily distinguished. On land or in shallow water, they can obscure each other; further, some may remain in the deep water when the main pod is resting.*

   While it is not possible to have an actual 'true' or 'accurate' number, our daily maximum number is probably as close as possible to the true number, given it relies on a number of estimates over each day when hippos are predominantly sedentary. There is perhaps only one way of accurately determining the true number and that is with marked individuals, but this is still not possible or effective with these dangerous animals. The use of drones was certainly effective in providing an estimate of numbers compared to the land counts, and could be used as an index of populations.

   We have revised the manuscript to reflect this response in the abstract, introduction, methods and discussion.

   Lines 38-40 in Abstract:
   "Use of a relatively low-cost drone provided a rigorous and repeatable method for estimating numbers and ages of hippos, ***other than in the early morning, compared to land counts, considered the most accurate method of counting hippos*** *~~but not in the early morning~~."*

   Line 52 in Introduction:
   "… but hippos are inherently difficult to count because ***of their aquatic lifestyle and behaviour*** *~~individuals regularly submerge and surface throughout the day and have uniform appearance~~."*

   Lines 61-69 in Introduction:
   *"**Even c**~~C~~ounts from land ~~or boats~~ **which** tend to be more accurate [4], given hippo pods can be observed for a long period of time,* ***cannot identify the true number of hippos in a pod, even a small one, without marked or recognised individuals. This is because hippos in the water continually surface and submerge, and individuals are not easily distinguishable [12].*** *~~allowing for differential submergence, but such counts can be dangerous and difficult or impossible to do where hippo pods are in remote or~~*

~~*not easily accessible areas [21,26].*~~ ***Accuracy improves when hippos rest in aggregations in shallow water ("rafting") [26] or on land, but still*** ~~*Further, line of sight from horizontal counts can be challenging, given*~~ *some individuals are inevitably obscured by others [27]*~~*, even though accuracy improves when hippos rest in aggregations, where most of their bodies are visible ("rafting") [28]*~~***. Although land counts are the most accurate method for counting hippos [2–4,28,29], they still have the potential to underestimate [30,31], and all counts should be considered a minimum, rather than a true count of hippos in an area [2,32,33].****"*

Lines 180-181 in Methods:
*"**The actual number of hippos in the lagoon on any particular day was unknown, and difficult to estimate given their behaviour, therefore true accuracy could not be calculated.**"*

Lines 305-310 in Discussion:
*"We showed that by flying a relatively cheap drone at 40 m, as long as not in the early morning, reasonable estimates of hippo numbers and demographics were achievable**. Although unable to estimate true accuracy for the drone counts, they were** equal to or better than land counts**, considered the most accurate method for counting hippos** [2–4,28,29]. **Both methods may still underestimate hippo numbers, which cannot be confirmed barring further testing where individuals can be recognised.**"*

Line 372 in Discussion:
*"Our analyses show that drone data can ~~provide accurate~~ **be used** to estimate~~s of~~ hippo pods …"*

3. On a personal note, you were very fortunate that the animals were not very bothered by the drone even at 40m. In my experience with horses and deer, animals appear to be very sensitive to the artificial buzz of a multirotor UAV. I wonder if perhaps the hippo's ear is not so sensitive to these sound frequencies (as compared to a horse, for example).
   Response: *Agreed. Interestingly, hippo hearing is believed to be good, and they communicate in frequencies (Barklow 1997) within the range of the Phantom 4. Perhaps their general lack of predation risk, particularly in the water, lowers their vigilance. Also, the study area was relatively close to an airstrip, so perhaps the hippos had become used to overhead sounds (although hippos in other areas also displayed minimal disturbance). We have revised the manuscript to address this:*

Lines 319-320 in Discussion:
*"Encouragingly, the drone did not disturb the hippos**, although the pod's proximity to an airstrip (1 km) may have habituated the hippos to aerial noises.**"*

Reviewer 1 comments

1. This is well executed research utilizing drone technology. As the technology develops to overcome the difficulties of drone control raised by authors in Discussion, this new techniques can be used widely in ecological monitoring.
   Response: *We appreciate this positive review and agree that drones have great potential.*

Reviewer 2 comments

1. My comments for the authors are contained in the attached document. The manuscript is fairly well written and easy to understand. The key weakness of the manuscript relates to the representation of the random variation in the data. As presented the models used do not adequately account for the potential total variation in the data.

Response: *Detailed responses are provided below, including additional analyses to address this key weakness.*

2. The first major problem with the manuscript is that the models for the statistical analyses of the data all under-represent the total variance. Mixed models are used for the analyses, which is fine. The fixed part of these models include Time, Height and their interactions. Time is the time of day when hippo counts were done and Height is the height above ground when hippo counts were carried out. This is also fine. The day of survey (day) is a random effect, presumably in all the models. This inadequately represents the random variation in the data. A complete and accurate representation of the random variation in the data should include
   (i) a random effect of Time*Day
   (ii) a random effect of Height*Day
   (iii) a random effect of Time*Height*Day
   (iv) Residual variance

   The model should thus be represented symbolically, as, for example,
   Model Count=Time Height Time*Height.
   Random Time*Day
   Random Height*Day
   Random Time*Height*Day

   Response: *To address this potential weakness, we reanalysed the data, as suggested by the reviewer, using the statistical package 'glmmTMB' (Brooks et al. 2017), which allows fitting more complex models, as well as allowing the addition of autocorrelation (an issue addressed further below). glmmTMB was specifically designed to deal with convergence issues, common to complex (generalized) linear mixed-effect models ([G]LMM) by working in the template model builder. Where convergence issues arose (see below), this likely indicates the model is simply too complex for the data available, and there are limited, if any, further options to fit them. The manuscript has been updated to reflect the use of glmmTMB.*

   > *Lines 167-170 in Methods:*
   > *"For model 1, we used a linear mixed model ~~(lmer and anova functions, lme4 package [52])~~, for model 2 a generalized linear mixed-effect model ~~(glmer function, lme4 package; Anova, car package [53])~~, with family Binomial and weights equal to the total number of hippos for each count, and for models 3 – 5 generalized linear mixed-effect models (~~manyglm and anova.manyglm functions, mvabund package [54])~~, with family ~~Negative Binomial~~Poisson, and a zero inflation variable."*
   >
   > *Lines 172-173 in Methods:*
   > *"**All modelling were conducted using the glmmTMB function (glmmTMB package [57]) ….**"*
   >
   > *Lines 175-177 in Methods:*
   > *"Differences among the levels of the effects were tested using post hoc pairwise comparisons, based on estimated marginal means, using a Tukey adjustment with the emmeans package [58] ~~(model 1 and 2) and the pairwise.comp function (manyglm package), adjusted for multiple testing using a step-down resampling procedure (models 3 -5)~~."*

   *We initially tried to include the random effects directly (e.g. Time*Day) in our original models however the models would not converge, as the variables (Time and Height) were set as categorical variables. Therefore, for inclusion as random effects, they were changed to numeric*

variables (e.g. for time: EM = 1, LM = 2, EA = 3, LA = 4), but for the fixed effects aspect of the model, they remained categorical. We tested the more complex models suggested by the reviewer and where these would not converge, we reduced them to see if simpler models could be fit (see Appendix 1 for full model output).

In summary, it was not possible to include all the additional sources of random variation, as suggested by reviewer 2, due to the complexity of these models (resulting in a lack of convergence). Where we included some of the variables and the models converged, these did not change our original results. The inclusion of these variables also prevented us from including the autocorrelation structures (see below). Given this, we did not include these additional variables in the models but acknowledge potential issues with the random effects structure in the manuscript.

> *Lines 165-167 in Methods:*
> ***"Attempts to include a more complex random effects structure led to inadequate convergence in the models and so we adopted a simple random effect structure."***
>
> *Lines 310-311 in Discussion:*
> ***"Further, the models used may inadequately represent the random variation in the data, and results could be improved with larger sample sizes."***

3. It is also well possible that the residual variance may be serially autocorrelated. This may be checked by allowing for a correlated residual variance, for example, by specifying an Ar(1) variance-covariance matrix and comparing this model with a model assuming uncorrelated residual variance.

<u>Response</u>*: Initially, we tested the presence of serial autocorrelation in residual variance on models fitted with the additional sources of random variation. We included the AR(1) covariance structure in accordance with the glmmTMB vignette. None of the models converged once the AR(1) structure was included (even the few models that had previously converged).*

We then reverted to the original simple model (i.e. with the random effect being (1|Day)) and included the AR(1) covariance structure. These models ran (except for Models 3-4 [juvenile and subadult counts]), and there was evidence of serial autocorrelation in the residuals of some models (Appendix 2) and so we have updated the manuscript to reflect these tests and results.

> *Lines 170-172 in Methods:*
> ***"We checked for serial autocorrelation in the residuals of all models by comparing models with AR(1) covariance structures to models assuming uncorrelated residuals, using likelihood ratio tests (LRT, anova function)."***
>
> *Lines 222-224 in Results:*
> ***"The inclusion of an AR(1) covariance structure did not improve model fit (χ^2(2) = 3.548, p = 0.170), so was omitted from the model."***
>
> *Lines 258-260 in Results:*
> ***"The inclusion of an AR(1) covariance structure significantly improved model fit (χ^2(2) = 18.760, p < 0.001), and was retained in the model."***
>
> *Lines 280-281 in Results:*
> ***"We were unable to include AR(1) covariance structures in the models due to lack of convergence."***

Lines 295-296 in Results:
"The inclusion of an AR(1) covariance structure significantly improved model fit ($\chi^2(2) = 6.228, p = 0.044$)."

4. Because the total sample size is rather small, 84, it may help to adjust the denominator degrees of freedom for t and F tests for bias using a Satterthwaite-type approximation, such as the Kenward-Rodger degrees of freedom.

Response: *The sample size was 112, as it included land counts, albeit still relatively small. To accurately test the significance of the effects in a GLMM ('GLMM FAQ' 2019;* https://bbolker.github.io/mixedmodels-misc/glmmFAQ.html*), the recommended order (from less recommended to more recommended) is Wald chi-square tests (e.g. car::Anova), likelihood ratio test (LRT, e.g. comparing full and reduced models via anova), and conditional F-tests with df correction (for LMMs only, e.g. Kenward-Roger). Therefore, we agree that adjusting the degrees of freedom (df) for F tests was a sound recommendation. Unfortunately, we could not find any R function/package to adjust df for GLMM models (or LMM models fit with glmmTMB); conditional F tests with df correction are relevant to LMMs only. In our original manuscript, for model 1 we used a conditional F-test with df correction (it is an LMM so this was possible), for model 2 we used a Wald chi-square test, and for models 3 – 5, we used LRTs. Given the above recommendations, we have revised the manuscript and used an LRT for model 2. Also, because model 1 is now fitted with glmmTMB, we had to use an LRT for this model also. In addition, Models 3-5 were re-specified using glmmTMB, and the resulting models checked with LRTs. The manuscript was updated accordingly:*

Lines 172-175 in Methods:
"All modelling was conducted using the glmmTMB function (glmmTMB package [57]), with the significance of the fixed effects, their interaction, and the random effect, determined by comparing full and reduced models using LRT (anova function)."

To investigate potential anti-conservatism in our results due to our sample size, we looked in detail at Model 1, given it's an LMM and therefore df can be adjusted (when the model is fitted with the lmer function) (Appendix 3). We found that the LRTs were slightly less conservative than the conditional F-test with df correction, but not unreasonably so. Given we have no option to implement conditional F-test with df correction for our GLMMs (or LMM fit with glmmTMB), we had to rely on the LRTs.

In summary, we could not adjust for small sample size on the glmmTMB models, due to limitations in statistical software. We made every effort to implement the changes where possible and have updated the manuscript where applicable. We believe our statistical tests are valid, and not significantly altered by the sample size. The change in the analyses and testing using LRTs did not significantly change any of our results for the fixed effects (e.g. nothing was significant that wasn't in the original and vice versa). There were some pairwise changes among the levels of the fixed effects which we revised in the text and graphs. Where changes have occurred in the results (lines 211-215, 249-255, 268-278, 288-295 and Supplementary information S2 Text), these do not significantly alter any of our interpretations. In summary,

- *For model 1, there were no important changes to the results.*
- *For model 2, the AR(1) covariance structure significantly improved model fit, rendering survey day insignificant as temporal patterns were incorporated in the AR(1) structure. Some posthoc test significances changed, but these did not affect our overall interpretations.*

- *For model 3, the difference in number of juveniles between land counts and drone counts at 40 m no longer fell below the significance level (but was close, p = 0.072), updated in the manuscript and plots.*
- *For model 4, the number of subadults counted from land counts and drone counts at 80 m was no longer below the significant level (but was close, p = 0.055), updated in the manuscript and plots.*
- *For model 5, the AR(1) covariance structure significantly improved model fit, rendering survey day insignificant as temporal patterns were incorporated in the AR(1) structure. Some posthoc test significances have changed, a notable one being that counts in the early morning at 120m are no longer significantly different from the land counts and counts at 40 m in the early morning.*

5. Are the land based counts included in the mixed model or are they not analysed together with the drone-based counts? This is not clear from the text.

   Response: *The land-based counts were included in all the models, along with the drone counts, so sample size was 112.We have clarified in the text:*

   Lines 159-160 in Methods:
   *"We tested the effect of **survey** height **(including land counts)**, time of day, and their interaction on variations in …"*

6. The assessment of the relative performance of the land based and drone-based methods at the various time and height combinations should be done relative to the known true value of hippos because all the methods may be biased. The manuscript compares the methods without reference to a benchmark or gold standard. This makes it impossible to determine whether or not a given method is better than its competitors. Since the lagoon had a fairly small hippo population and the population was counted many times it should be possible to get a reliable estimate of the total population size that can be used as a reference. If all the methods are biased downwards, for example, then even the maximum cannot be used as a benchmark for assessing accuracy.

   Response: *An overall estimate (from the 112 counts) could be calculated but this assumes no immigration/emigration from waterbodies. However, movement between waterbodies does occur, as indicated by our data. Hippos do not always return to the same lagoon each morning, particularly when water is drying back, as during the months of our data collection (lines 369-370). However, they are unlikely to move between lagoons during the day, and it was because of this that we restricted ourselves to use of daily maximum. We have updated the manuscript to provide these daily maximum counts, clarifying that one overall hippo estimate was not possible. In addition, see response to editor comments for other changes regarding the issue with comparing drone and land counts.*

   Lines 177-181 in Methods:
   *"We determined the maximum number of hippos seen for each day (from any drone or land count), ~~as a proxy of potential true number of hippos,~~ investigating how counts compared to this daily maximum, as another measure of ~~true~~ accuracy, given that hippos generally do not move out of lagoons during diurnal hours [56]. **The actual number of hippos in the lagoon on any particular day was unknown, and difficult to estimate given their behaviour, therefore true accuracy could not be calculated.**"*

   Lines 196-198 in Results:
   "We use daily maximum counts to describe the population because of potential emigration and immigration between surveys days: first two days (14 hippos), following two days (12 hippos), following two days (10 hippos), and last survey day (9 hippos)."

Line 232 in Results:
"Our daily maximum counts~~, a proxy of potential true number of hippos in the lagoon,~~ occurred at all times of the …"

Figure 3 caption:
"Relationship between maximum hippo counts ~~(a proxy of the true count)~~ and …"

7. Lines 102-114. The authors should state clearly how many surveys were carried out at each height and time combination per day? This does not come out clearly. Over how many days were the drone surveys carried out?
   Response: *This is now clarified:*

   Line 88-91 in Methods:
   "We conducted land and drone counts over a lagoon (-19.41725°E, 22.56815°S, 2.4 ha), with a resident hippo population, within NG26 (Abu Concession) of the Okavango Delta, northern Botswana (Fig. 1), ***over seven days (****on 7th, 8th, 11th, 13th and 14th November 2017 and 2nd and 3rd December 2017****)****."*

   Line 117-120 in Methods:
   *"****This resulted in 12 drone surveys per day (one at each height [120 m, 80 m, 40 m] at each time of day [EM, LM, EA, LA]), for seven days. Therefore,*** *we completed a total of 84 drone counts (28 per height, 21 per* ***time of*** *day)."*

   Line 127-129 in Methods:
   *"****Therefore,*** *we completed* ***four land counts per day (EM, LM, EA, LA), for seven days, resulting in*** *a total of 28 land counts ~~(seven per day)~~****. In conjunction with the drones counts, this equated to a total of 112 surveys.****"*

8. Line 119 states that 4 land counts were done per day but line 121 says that 7 surveys were done per day? Which is which?
   Response: *This was an error. There were four counts per day, over seven days. The section has been amended to correct this (see above).*

9. Line 133. Insert "and" between ..<184 cm and less than….
   Response: *Corrected*

10. Line 156. Survey date should not be used as a random effect. Instead, use the random effects outlined in (1) above.
    Response: *Discussed above.*

11. Lines 164-165. The maximum number of hippos seen from any count cannot be used as benchmark for assessing accuracy of all the contending methods since it varies with the methods and is not the true number of hippos in the lagoon. The true population size of hippos in the lagoon is needed to assess accuracy as the difference between the estimated population size and the true population size.
    Response: *Discussed above (in response to Editor's comments).*

12. Line 197. It is not clear how hippo population size can be significantly related to a random effect? Were there actually 35.7% fewer hippos or 35.7% fewer hippos were estimated at the end of the survey? The former would mean that the true population size of hippos in the lagoon was known and so can be used as the benchmark.

Response: *Hippo count was significantly related to the random effect survey day (determined by comparing models with and without this variable), as over the course of the surveys there were less hippos counted (discussed above). We did not know the true number of hippos, the 35.7% referenced in the manuscript is comparing maximum count from the first day of survey to the last day.*

Line 214 in Results:
*"… **fewer hippos were counted in subsequent days of the survey** ~~there were 35.7% fewer hippos at the end of the survey, compared to the beginning of the surveys~~."*

13. Lines 199-206. The methods should not just be compared to each other but also with the true population size as the gold standard.
    Response: *Discussed above (in response to Editor's comments)*

14. Lines 338-339. How would such a recommendation be implemented? Would this require first studying the behaviour of hippos before counting them to establish when hippos are resting. In many places, this may not be feasible.
    Response: *We agree that extensive behaviour studies are not feasible in many areas or every time a survey needs to be conducted. But having a general knowledge of hippo activity patterns for a country/region could be obtained by conducting a few observations during the day, or by asking local people the general behaviour of hippos in the area. The idea is not to conduct a full investigation for each pod before surveying but having a general idea of what the hippos are doing in that region at that time of year. We have updated this section with suggestions how this recommendation could be achieved, but also acknowledging that this will not always be feasible.*

    Line 365-368 in Discussion:
    *"Hippo behaviour may differ by region or habitat, we therefore recommend adapting the timing of surveys to when hippo are resting, which may vary in location and time, **and could be determined through simple observations, examining existing literature, or local knowledge where possible**."*

15. Line 344. Provide instead of provides
    Response: *Corrected*

Additional requirements

1. Please ensure that your manuscript meets PLOS ONE's style requirements, including those for file naming.
   Response: *Thank you for linking to these style templates. In accordance with the templates, some small formatting changes have been made in the text (not detailed here).*

2. We note that Figure 1 in your submission contains map/satellite images which may be copyrighted. We require you to either (a) present written permission from the copyright holder to publish these figures specifically under the CC BY 4.0 license, or (b) remove the figures from your submission ….
   Response: *We have drawn another map using our drone imagery.*

   Figure 1 caption:
   *"Lagoon (shaded yellow) where hippos were counted in NG26, within the Okavango Delta in Botswana, showing where the drone was launched and land counts were done. **Note: background is an orthomosaic created using drone images.**"*

3. In your Methods section, please provide additional location information of the study area, including geographic coordinates for the data set if available.

   Response: *In the original manuscript we had provided the geographic co-ordinates in the Methods "… over a lagoon (-19.41725°E, 22.56815°S, 2.4 ha)…". Given this must not have been clear enough, we have made the first section of the methods detailing the study site a separate paragraph (moving the drone specifications to the Methods section 'Drone Surveys'). If this is not sufficient, please specify what additional information is required.*

4. We note that you have indicated that data from this study are available upon request. PLOS only allows data to be available upon request if there are legal or ethical restrictions on sharing data publicly. In your revised cover letter, please address the following prompts:

   a) If there are ethical or legal restrictions on sharing a de-identified data set, please explain them in detail (e.g., data contain potentially sensitive information, data are owned by a third-party organization, etc.) and who has imposed them (e.g., an ethics committee). Please also provide contact information for a data access committee, ethics committee, or other institutional body to which data requests may be sent.

   Response: *Apologies for not previously specifying why the raw data could not be made available. The raw data (drone videos) cannot be made publicly available in accordance with the University of New South Wales' animal care and ethics committee, which require permission be sought prior to any moving image being shown outside the university. The relevant ethics committee to contact is UNSW's Animal Care & Ethics Committee, contact via animalethics@unsw.edu.au, quoting ACEC Number 17/75A.*

   b) If there are no restrictions, please upload the minimal anonymized data set necessary to replicate your study findings as either Supporting Information files or to a stable, public repository and provide us with the relevant URLs, DOIs, or accession numbers.

   Response: *Whilst, we cannot upload the raw data, the minimal data set necessary to replicate the results of the study is included in the manuscript via supplementary information Table 1.*

5. Please amend your list of authors on the manuscript to ensure that each author is correctly linked to each affiliation. Authors' affiliations should reflect the institution where the work was done (if authors moved subsequently, you can also list the new affiliation stating "current affiliation:…." as necessary).

   Response: *We have reviewed this and the details are now correct.*

References

Barklow, W.E. Some underwater sounds of the hippopotamus (*Hippopotamus amphibius*). Marine and Freshwater Behaviour and Physiology. 1997;29:237–249.

Brooks ME, Kristensen K, van Benthem KJ, Magnusson A, Berg CW, Nielsen A, et al. glmmTMB balances speed and flexibility among packages for zero-inflated generalized linear mixed modeling. R J. 2017;9(2):378–400.

Delvingt W. Ecologie de l'hippopotame (*Hippopotamus amphibius* L.) au Parc National des Virunga (Zaïre). PhD Thesis, Faculté des sciences agronomiques de l'état Gembloux. 1978.

Klingel H. Hippopotamus amphibius Common Hippopotamus. In: Kingdon J, Hoffman M, editors. Mammals of Africa: Volume VI: Pigs, Hippopotamuses, Chevrotain, Giraffes, Deer and Bovids. London: Bloomsbury Publishing; 2013. p. 68–77.

Appendix 1 - Additional information in response to reviewer 2's comment #2

Model 1 (effect of height and time of day on total hippo count)

Original_model <- glmmTMB(total ~ height*time + (1|day))

Model_i <- glmmTMB(total ~ height*time + (time_numeric|day))

This model ran and we compared it to the original model, finding no difference (large p value), and the more complex model had a slightly higher AIC.

anova(Original_model, Model_i)

	Df	AIC	BIC	logLik	dev	Chisq	Df	Pr(>Chisq)
Original_model	18	467.64	516.58	-215.82	431.64			
Model_i	20	470.19	524.56	-215.09	430.19	1.460	2	0.482

Other complex models tried
Model_iii_int <- glmmTMB(total ~ height*time + (height_numeric*time_numeric|day))
Model_iii_add <- glmmTMB(total ~ height*time + (height_numeric + time_numeric|day))
Model_ii <- glmmTMB(total ~ height*time + (height_numeric|day))

None of these models converged.

Model 2 (effect of height and time of day on percentage of hippos assigned to age classes)

Original_model <- glmmTMB(percentage ~ height*time + (1|day), weights=total, family=binomial)

Model_iii_add <- glmmTMB(percentage ~ height*time+(height_numeric+time_numeric|day),
weights=total, family=binomial)

This model ran and we compared it to the original model, finding no difference (large p value), and the more complex model had a slightly higher AIC.

anova(Original_model, Model_iii_add)

	Df	AIC	BIC	logLik	dev	Chisq	Df	Pr(>Chisq)
Original_model	17	463.07	509.29	-214.54	429.07			
Model_iii_add	22	466.68	526.49	-211.34	422.68	6.391	5	0.270

Other complex models tried
Model_iii_int <- glmmTMB(percentage ~ height*time+(height_numeric*time_numeric|day),
weights=total, family=binomial)

This model would not converge.

Models 3 and 4 (effect of height and time of day on number of juveniles and subadults)

Original_model <- glmmTMB(juvenile ~ height*time + (1|day), ziformula=~1, family=poisson)

Complex models tried
Model_iii_int <- glmmTMB(juvenile ~ height*time + (height_numeric*time_numeric|day) ,
ziformula=~1, family=poisson)

Model_iii_add <- glmmTMB(juvenile ~ height*time + (height_numeric + time_numeric|day) , ziformula=~1, family=poisson)
Model_ii <- glmmTMB(juvenile ~ height*time + (height_numeric|day) , ziformula=~1, family=poisson)
Model_i <- glmmTMB(juvenile ~ height*time + (time_numeric|day) , ziformula=~1, family=poisson)

None of these models converged. Results for subadults are not shown, but were the same.

Model 5 (effect of height and time of day on number of adults)

Original_model <- glmmTMB(adult ~ height*time + (1|day) , ziformula=~1, family=poisson)

Model_i <- glmmTMB(adult ~ height*time + (time_numeric|day) , ziformula=~1, family=poisson)

This model ran and we compared it to the original model, finding no difference (large p value), and the more complex model had a slightly higher AIC.

anova (Original_model, Model_i)

	Df	AIC	BIC	logLik	dev	Chisq	Df	Pr(>Chisq)
Original	18	456.49	505.42	-210.25	420.49			
Model_i	20	459.05	513.42	-209.52	419.05	1.447	2	0.485

Other complex models tried
Model_iii_int <- glmmTMB(adult ~ height*time + (height_numeric*time_numeric|day) , ziformula=~1, family=poisson)
Model_iii_add <- glmmTMB(adult ~ height*time + (height_numeric + time_numeric|day) , ziformula=~1, family=poisson)
Model_ii <- glmmTMB(adult ~ height*time + (height_numeric|day) , ziformula=~1, family=poisson)

None of these models converged.

Appendix 2 - Additional information in response to reviewer 2's comment #3

Model 1 (effect of height and time of day on total hippo count)

Original_model <- glmmTMB(total ~ height*time + (1|day))

Model_iv <- glmmTMB(total ~ height*time + (1|day) + ar1(times + 0 | group))

This model ran and we compared it to the original model, finding no difference (large p value), and the more complex model had a slightly higher AIC. However, we have incorporated the more complex model suggested by the reviewer.

anova(Original_model, Model_iv)

	Df	AIC	BIC	logLik	dev	Chisq	Df	Pr(>Chisq)
Original_model	18	467.64	516.58	-215.82	431.64			
Model_iv	20	468.10	522.47	-214.05	428.10	3.548	2	0.170

Other complex models tried

Model_iii_int <- glmmTMB(total ~ height*time + (height_numeric*time_numeric|day) + ar1(times + 0 | group))

Model_iii_add <- glmmTMB(total ~ height*time + (height_numeric + time_numeric|day) + ar1(times + 0 | group))

Model_ii <- glmmTMB(total ~ height*time + (height_numeric|day) + ar1(times + 0 | group))

Model_i <- glmmTMB(total ~ height*time + (time_numeric|day) + ar1(times + 0 | group))

None of these models converged.

Model 2 (effect of height and time of day on percentage of hippos assigned to age classes)

Original_model <- glmmTMB(percentage ~ height*time + (1|day), family=binomial, weights=total)

Model_iv <- glmmTMB(percentage ~ height*time + (1| day) + ar1(times + 0 | group), family=binomial, weights=total)

This model ran and we compared it to the original model. The model with the AR(1) covariance structure was significantly better (small p value and lower AIC), indicating residuals were serially autocorrelated. We have included the more complex model in the manuscript.

anova(Original_model, Model_iv)

	Df	AIC	BIC	logLik	dev	Chisq	Df	Pr(>Chisq)
Original_model	17	463.07	509.29	-214.54	429.07			
Model_iv	19	448.31	499.97	-205.16	410.31	18.76	2	<0.001

Other complex models tried

Model_iii_int <- glmmTMB(percentage ~ height*time + (height_numeric*time_numeric|day) + ar1(times + 0 | group), family=binomial, weights=total)

Model_iii_add <- glmmTMB(percentage ~ height*time + (height_numeric + time_numeric|day) + ar1(times + 0 | group), family=binomial, weights=total)

Model_ii <- glmmTMB(percentage ~ height*time + (height_numeric|day) + ar1(times + 0 | group), family=binomial, weights=total)

Model_i <- glmmTMB(percentage ~ height*time + (time_numeric|day) + ar1(times + 0 | group), family=binomial, weights=total)

None of these models converged.

Model 3 and 4 (effect of height and time of day on number of juveniles and subadults)

Original_model <- glmmTMB(juveniles ~ height*time + (1|day), ziformula=~1, family=poisson)

Complex models tried

Model_iv <- glmmTMB(juveniles ~ height*time + (1|day) + ar1(times + 0 | group) , ziformula=~1, family=poisson)

Model_iii_int <- glmmTMB(juveniles ~ height*time + (height_numeric*time_numeric|day) + ar1(times + 0 | group), ziformula=~1, family=poisson)

Model_iii_add <- glmmTMB(juveniles ~ height*time + (height_numeric + time_numeric|day) + ar1(times + 0 | group), ziformula=~1, family=poisson)

Model_ii <- glmmTMB(juveniles ~ height*time + (height_numeric|day) + ar1(times + 0 |group), ziformula=~1, family=poisson)

Model_i <- glmmTMB(juveniles ~ height*time + (time_numeric|day) + ar1(times + 0 | group), ziformula=~1, family=poisson)

None of these models converged. Results for subadults are not shown, but were the same.

Model 5 (effect of height and time of day on number of adults)

Original_model <- glmmTMB(adults ~ height*time + (1|day), ziformula=~1, family=poisson)

Model_iv <- glmmTMB(adults ~ height*time + (1|day) + ar1(times + 0 | group), ziformula=~1, family=poisson)

This model ran and we compared it to the original model. The model with the AR(1) covariance structure was slightly better (significant p value and slightly lower AIC), indicating residuals may be serially autocorrelated. We have included the more complex model in the manuscript.

anova(Original_model, Model_iv)

	Df	AIC	BIC	logLik	dev	Chisq	Df	Pr(>Chisq)
Original_model	18	456.49	505.42	-210.25	420.49			
Model_iv	20	454.26	508.63	-207.13	414.26	6.228	2	0.044

Other complex models tried

Model_iii_int <- glmmTMB(adults ~ height*time + (height_numeric*time_numeric|day) + ar1(times + 0 | group), ziformula=~1, family=poisson)

Model_iii_add <- glmmTMB(adults ~ height*time + (height_numeric + time_numeric|day) + ar1(times + 0 | group), ziformula=~1, family=poisson)

Model_ii <- glmmTMB(adults ~ height*time + (height_numeric|day) + ar1(times + 0 | group), ziformula=~1, family=poisson)

Model_i <-glmmTMB(adults ~ height*time + (time_numeric|day) + ar1(times + 0 | group), ziformula=~1, family=poisson)

None of these models converged.

Appendix 3 - Additional information in response to reviewer 2's comment #4

Model 1 (effect of height and time of day on total hippo count)

Original_model <- lmer(total ~ height*time + (1|day))

In the original manuscript, the model was run with a Satterthwaite's method of ddf adjustment.

anova(Original_model)

Type III Analysis of Variance Table with Satterthwaite's method						
	Sum sq	Mean sq	NumDF	DenDF	F value	Pr(>F)
height	32.786	10.929	3	90	4.108	0.009
time	117.643	39.214	3	90	14.741	<0.001
height*time	27.143	3.016	9	90	1.134	0.348

We reran the original model with Kenward-Roger's method of adjustment, and there was no change.

anova(Original_model, ddf="Kenward-Roger")

Type III Analysis of Variance Table with Kenward-Roger's method						
	Sum sq	Mean sq	NumDF	DenDF	F value	Pr(>F)
height	32.786	10.929	3	90	4.108	0.009
time	117.643	39.214	3	90	14.741	<0.001
height*time	27.143	3.016	9	90	1.134	0.348

Compare these results to those of the LRT on the glmmTMB models in the revised manuscript.

glmmTMB_full <- glmmTMB(total ~ height*time + (1|day))

Effect of height*time
glmmTMB_add <- glmmTMB(total ~ height + time + (1|day))
anova(glmmTMB_full, glmmTMB_add)

	Df	AIC	BIC	logLik	dev	Chisq	Df	Pr(>Chisq)
glmmTMB_full	9	460.92	485.39	-221.46	442.92			
glmmTMB_add	18	467.64	516.58	-215.82	431.64	11.276	9	0.257

Effect of height
glmmTMB_height <- glmmTMB(total ~ time + (1|day))
anova(glmmTMB_add, glmmTMB_height)

	Df	AIC	BIC	logLik	dev	Chisq	Df	Pr(>Chisq)
glmmTMB_add	6	467.10	483.41	-227.55	455.10			
glmmTMB_height	9	460.92	485.39	-221.46	442.92	12.180	3	0.007

Effect of time
glmmTMB_time <- glmmTMB(total ~ height + (1|day))
anova(glmmTMB_add, glmmTMB_ time)

	Df	AIC	BIC	logLik	dev	Chisq	Df	Pr(>Chisq)
glmmTMB_add	6	493.30	409.61	-240.65	481.30			
glmmTMB_time	9	460.92	485.39	-221.46	442.92	38.384	3	<0.001
