# Supplementary material for: Drone-based effective counting and ageing of hippopotamus (Hippopotamus amphibius) in the Okavango Delta in Botswana
Source: PLoS One. 2019 Dec 5;14(12):e0219652. doi: 10.1371/journal.pone.0219652 (PMC6894862; doi:10.1371/journal.pone.0219652)
Supplement: Attachment — Submitted filename: Review_Hippo surveys.docx [file pone.0219652.s004.docx]

**Review of Drone-based effective counting and ageing of hippopotamus in the Okavango Delta in Botswana by Inman et al.**

This manuscript reports results of counting hippos in a small lagoon in the Okavango Delta in Botswana. Hippos were counted using land based and drone-based counts at 40 m, 80 m and 120. For each height counts were carried out at four times of day.

I find three key problems with this manuscript, which if addressed would make it stronger.

1) The first major problem with the manuscript is that the models for the statistical analyses of the data all under-represent the total variance. Mixed models are used for the analyses, which is fine. The fixed part of these models include Time, Height and their interactions. Time is the time of day when hippo counts were done and Height is the height above ground when hippo counts were carried out. This is also fine. The day of survey (day) is a random effect, presumably in all the models. This inadequately represents the random variation in the data. A complete and accurate representation of the random variation in the data should include
(i) a random effect of Time*Day
(ii) a random effect of Height*Day
(iii) a random effect of Time*Height*Day
(iv) Residual variance

The model should thus be represented symbolically, as, for example,

Model Count=Time Height Time*Height.

Random Time*Day

Random Height*Day

Random Time*Height*Day

It is also well possible that the residual variance may be serially autcorrelated. This may be checked by allowing for a correlated residual variance, for example, by specifying an Ar(1) variance-covariance matrix and comparing this model with a model assuming uncorrelated residual variance.

Are the land based counts included in the mixed model or are they not analysed together with the drone-based counts? This is not clear from the text.

2) Because the total sample size is rather small, 84, it may help to adjust the denominator degrees of freedom for t and F tests for bias using a Satterthwaite-type approximation, such as the Kenward-Rodger degrees of freedom.
3) The assessment of the relative performance of the land based and drone-based methods at the various time and height combinations should be done relative to the known true value of hippos because all the methods may be biased. The manuscript compares the methods without reference to a benchmark or gold standard. This makes it impossible to determine whether or not a given method is better than its competitors. Since the lagoon had a fairly small hippo population and the population was counted many times it should be possible to get a reliable estimate of the total population size that can be used as a reference. If all the methods are biased downwards, for example, then even the maximum cannot be used as a benchmark for assessing accuracy.

Minor comments

4) Lines 102-114. The authors should state clearly how many surveys were carried out at each height and time combination per day? This does not come out clearly. Over how many days were the drone surveys carried out?
5) Line 119 states that 4 land counts were done per day but line 121 says that 7 surveys were done per day? Which is which?
6) Line 133. Insert “and” between ..<184 cm and less than…
7) Line 156. Survey date should not be used as a random effect. Instead, use the random effects outlined in (1) above.
8) Lines 164-165. The maximum number of hippos seen from any count cannot be used as benchmark for assessing accuracy of all the contending methods since it varies with the methods and is not the true number of hippos in the lagoon. The true population size of hippos in the lagoon is needed to assess accuracy as the difference between the estimated population size and the true population size.
9) Line 197. It is not clear how hippo population size can be significantly related to a random effect?
10) Were there actually 35.7% fewer hippos or 35.% fewer hippos were estimated at the end of the survey? The former would mean that the true population size of hippos in the lagoon was known and so can be used as the benchmark.
11) Lines 199-206. The methods should not just be compared to each other but also with the true population size as the gold standard.
12) Lines 338-339. How would such a recommendation be implemented? Would this require first studying the behaviour of hippos before counting them to establish when hippos are resting. In many places, this may not be feasible.
13) Line 344. Provide instead of provides.
